# Retention, Fasting Patterns, and Weight Loss With an Intermittent Fasting App: Large-Scale, 52-Week Observational Study

**DOI:** 10.2196/35896

**Published:** 2022-10-04

**Authors:** Luisa Torres, Joy L Lee, Seho Park, R Christian Di Lorenzo, Jonathan P Branam, Shelagh A Fraser, Benjamin A Salisbury

**Affiliations:** 1 LifeOmic Indianapolis, IN United States; 2 Indiana University School of Medicine Indianapolis, IN United States; 3 The Regenstrief Institute Indianapolis, IN United States; 4 Infinia ML Durham, NC United States; 5 Priority Physicians Carmel, IN United States; 6 Program in Biomedical Sciences, Wright State University Dayton, OH United States

**Keywords:** intermittent fasting, time-restricted eating, weight loss, obesity, mobile apps, diet trackers, retention

## Abstract

**Background:**

Intermittent fasting (IF) is an increasingly popular approach to dietary control that focuses on the timing of eating rather than the quantity and content of caloric intake. IF practitioners typically seek to improve their weight and other health factors. Millions of practitioners have turned to purpose-built mobile apps to help them track and adhere to their fasts and monitor changes in their weight and other biometrics.

**Objective:**

This study aimed to quantify user retention, fasting patterns, and weight loss by users of 2 IF mobile apps. We also sought to describe and model starting BMI, amount of fasting, frequency of weight tracking, and other demographics as correlates of retention and weight change.

**Methods:**

We assembled height, weight, fasting, and demographic data of adult users (ages 18-100 years) of the LIFE Fasting Tracker and LIFE Extend apps from 2018 to 2020. Retention for up to 52 weeks was quantified based on recorded fasts and correlated with user demographics. Users who provided height and at least 2 readings of weight and whose first fast and weight records were contemporaneous were included in the weight loss analysis. Fasting was quantified as extended fasting hours (EFH; hours beyond 12 in a fast) averaged per day (EFH per day). Retention was modeled using a Cox proportional hazards regression. Weight loss was analyzed using linear regression.

**Results:**

A total of 792,692 users were followed for retention based on 26 million recorded fasts. Of these, 132,775 (16.7%) users were retained at 13 weeks, 54,881 (6.9%) at 26 weeks, and 16,478 (2.1%) at 52 weeks, allowing 4 consecutive weeks of inactivity. The survival analysis using Cox regression indicated that retention was positively associated with age and exercise and negatively associated with stress and smoking. Weight loss in the qualifying cohort (n=161,346) was strongly correlated with starting BMI and EFH per day, which displayed a positive interaction. Users with a BMI ≥40 kg/m^2^ lost 13.9% of their starting weight by 52 weeks versus a slight weight gain on average for users with starting BMI <23 kg/m^2^*.* EFH per day was an approximately linear predictor of weight loss. By week 26, users lost over 1% of their starting weight per EFH per day on average. The regression analysis using all variables was highly predictive of weight change at 26 weeks (*R*^2^=0.334) with starting BMI and EFH per day as the most significant predictors.

**Conclusions:**

IF with LIFE mobile apps appears to be a sustainable approach to weight reduction in the overweight and obese population. Healthy weight and underweight individuals do not lose much weight on average, even with extensive fasting. Users who are obese lose substantial weight over time, with more weight loss in those who fast more.

## Introduction

### Background

Worldwide, 13% of adults have obesity (BMI ≥30 kg/m^2^) and 39% are overweight (BMI ≥25 kg/m^2^) [1]. In the United States, obesity is >3 times higher at 42.5% of the adult population [2], and by 2030, the prevalence is expected to be close to 50% [3]. Mobile health apps that incorporate practices such as intermittent fasting (IF) may be a cost-effective approach to mitigating weight gain.

IF is a set of dietary patterns commonly pursued for weight loss that limits the timing of eating without restricting food content. Studies have shown that various IF methods are effective for weight loss in people who are overweight [4-9], including time-restricted eating, alternate day fasting, and a 5:2 diet [10-14]. However, these studies have been conducted in small populations (<200 completers), for short durations (a few weeks up to 6 months), and with narrowly defined IF protocols assigned to participants. In the real-world setting, IF patterns may not be as cleanly defined, especially over longer durations during which multiple fasting patterns may be explored.

Mobile apps for IF and weight tracking offer an opportunity to examine IF in a less-controlled setting and investigate its real-world efficacy for weight management. They are also a low-cost intervention for addressing obesity in the general population and may incentivize the adoption of healthy habits, including exercise and healthy eating [15,16]. Despite their potential benefits, the use of mobile health apps has been limited owing to low retention rates [17-19], and only a handful of available health apps have been subjected to rigorous study to establish their efficacy.

### This Study

We evaluated retention, fasting patterns, and weight change among users of 2 free IF tracking apps, collectively known as LIFE apps: LIFE Fasting Tracker (LFT), which is focused on fasting, and LIFE Extend (LX), which additionally supports tracking of physical activity, mindfulness, sleep, and healthy plant intake. From 2018 to 2020, the 2 apps acquired a combined user base of 2.5 million downloads. User accounts and backend data storage for the 2 apps are shared, such that fasts could be started in one app and stopped in the other, and all the data are interchangeable. LFT was launched over a year earlier than LX, so only a small fraction of the data in this study was generated via LX.

We followed nearly 800,000 users for retention and real-world fasting behaviors. We further analyzed weight change patterns relative to app use and demographics in a subpopulation of over 160,000 users who used apps to track their weight over time.

We showed that practicing IF with a dedicated mobile app is an effective and sustainable approach to weight loss in individuals initially classified as overweight and obese. Many users consistently used the apps to record fasts every week for months. Users with obesity lost substantial weight over time, with more weight loss in avid fasters. Weight loss in users with obese or overweight BMI was sustained, on average, for up to a year with little rebound. Conversely, users with a healthy or underweight BMI lost little or no weight, even with extensive fasting over 52 weeks.

## Methods

### Mobile Apps and Users

We assembled all fasting and weight data for users who began using either of the LIFE apps between the launch of the LFT in May 2018 and December 2020. Analyses of fasting, retention, and weight are all relative to when the user began recording fasts in the apps, minimizing seasonal and other calendar effects. Other voluntary data collected were sex, age, race, height, diet, exercise frequency, stress level, smoking status, and primary health concern. For inclusion in our analyses, users had to have provided sex and date of birth and recorded at least one fast, the first of which had to have been started on or after their 18th birthday.

For the weight change analyses, we further required the user to have provided height and an initial weight recorded within 7 days of the first fast. Heights and weights had to have been within validation ranges of 145-203 cm and 25-249 kg, respectively. Height and weight can be entered in either metric or imperial units, with subsequent conversion to metric units for storage and analysis. We identified 902 users whose weight change at weeks 1 to 52 was >5 SD from the average across all users for that week. Without knowing which value or values were presumably misentered, we simply excluded those users entirely from the weight analysis, made feasible by the study’s large sample size. Weights were subject to a 24-hour *burn-in* period, using the last weight recorded during that time as the baseline value. This burn-in accommodated users who may have entered an initial weight in the app based on their recollection and entered an update after checking it on a scale or who corrected their entry after checking units.

### Fasting

We assembled all fasting records for the full set of nearly 800,000 users. Although the apps allowed shorter and longer fasts to be tracked, we eliminated fasts under 8 hours and truncated fasts to a maximum length of 240 hours. To reduce the effects of forgotten fasts that were ended and saved in the apps long after eating had resumed, we eliminated any fast that was 120 hours or longer but where a fasting goal of under 24 hours had been specified by the user. This yielded 25,983,817 fasts for our analyses.

We aggregated fasting statistics for each user for weeks 1 to 104 but primarily investigated weeks 1 to 52. Information regarding week 53 to 104 was used, when available and applicable, to determine retention. For each week, we totaled the number of started fasts, the average fast length, and the sum of hours beyond the first 12 in a fast, which we call extended fasting hours (EFH), and EFH per day (ie, EFH per day=sum of EFH for all fasts started in a week/7). The 12-hour time point in a fast is when the body is expected to have depleted the energy from recently consumed food and may begin a metabolic switch to deriving energy from the breakdown of fat [20]. This shift is also referred to as entering ketosis and is thought to help drive weight loss and other health benefits [20]. EFH per day also presents a unified way to analyze fasting effects for people with different total fasting time and frequency but similar time in ketosis. For example, a user who performs daily 16-hour fasts will have the same 4 EFH per day as a user who performs two 26-hour fasts per week, even though their total recorded fasting time is quite different (112 vs 52 hours per week).

We also calculated the cumulative means of these measures for all weeks, up to and including the given week.

### Retention

We assessed user retention based solely on records of completed fasts and not on other user behavior such as log-ins or use of other app features. Starting with the date of each user’s first fast, we assessed their fasting activity for each week. The most restrictive definition of retention is when a user is only considered retained so long as they record a fast in each consecutive week. We refer to this definition as retention with a 0-week grace period. In contrast, the most lenient definition of retention is where the user is considered retained the entire time between their first and last recorded fast, regardless of how much activity they have in between. We refer to this as retention with an unlimited grace period. This definition is also sometimes called rolling retention [21].

We explored retention by varying the number of weeks in the grace period. We looked at 0, 2, 4, 8, 13, 26, and unlimited-week grace periods. After considering this spectrum of retention metrics, we decided to apply the 4-week grace period retention definition for all subsequent analyses. For example, if the user recorded no fasts in weeks 10 to 13 but did fast in week 14, the user was still considered retained in weeks 10 to 14, but if they resumed fasting in week 15 or later, their retention would have ended with week 9. Note that our univariate estimates of retention are conservative because many users start near the end of our data collection period, thus not having the opportunity to be counted as active in the app during the full 52 weeks (plus the grace period) that they might otherwise have counted toward. In the multivariate analysis, we used right censoring to account for this issue.

### Weight Change and BMI

Users were included in the weight change analyses for all weeks for which they satisfied the 4-week grace period retention definition and in which they had a recorded weight. To account for the wide range of starting weights, weight change was analyzed as percent change from the user’s starting weight. The effect of obesity was also considered in some analyses by stratification on starting BMI. We categorized BMI using the Centers for Disease Control and Prevention definitions [22], with the further division of the healthy BMI category into healthy low and healthy high defined as the ranges 18.5 to 22 and 23 to 24, respectively. Healthy low and healthy high categories had an approximately equal representation in our baseline user data.

For the weekly weight aggregates, we calculated mean weight and the number of weights recorded during the week. The baseline weights were excluded from the week 1 aggregates.

### Analysis

We performed all data analysis using Python 3.9 libraries in a JupyterLab [23] notebook environment installed within LifeOmic’s Precision Health Cloud, the secure, Health Insurance Portability and Accountability Act–compliant backend of the LIFE apps. A security review process was used to ensure that no identifiable data were released from the precision health cloud. In addition to providing descriptive summary statistics, including means, SDs, and percentages, we used multivariate modeling approaches. Retention was modelled using Cox proportional hazards regression, as implemented in the Lifelines package (version 0.27.1) [24]. Right censoring was applied to users who joined late in the study and did not have the opportunity to be retained for 52 weeks. Weight change was modeled using ordinary least squares regression, as implemented in Statsmodels (version 0.12.2) [25]. Graphs were generated in Seaborn (version 0.11.1) [26], which was also used to generate the CIs displayed, except for the hazard ratios and regression figures, which were generated in Plotly (version 5.0.0) [27]. Data handling was managed using Pandas (version 1.3.1) [28].

### Ethical Considerations

This study was exempt from institutional review board approval per Indiana University’s research guidelines [29]. The study consisted of retrospective secondary analysis of deidentified data. The use of these data for research and aggregate reporting is covered in the privacy policy of the LIFE apps [30].

## Results

### LIFE Apps Users

A total of 792,692 users satisfied the inclusion requirements for the fasting and retention analysis. The detailed demographic and biometric data for this population are presented in Multimedia Appendix 1. Their mean age was 36.7 (SD 10.9, range 18-100) years, and 81.3% of users were female. Users were located in nearly 200 different countries, with the majority being in the United States. Of these, 161,346 users met the height and weight measurement requirements and recorded at least one post–burn-in weight. This subpopulation was demographically similar to the entire population.

### Retention

**Figure 1:**
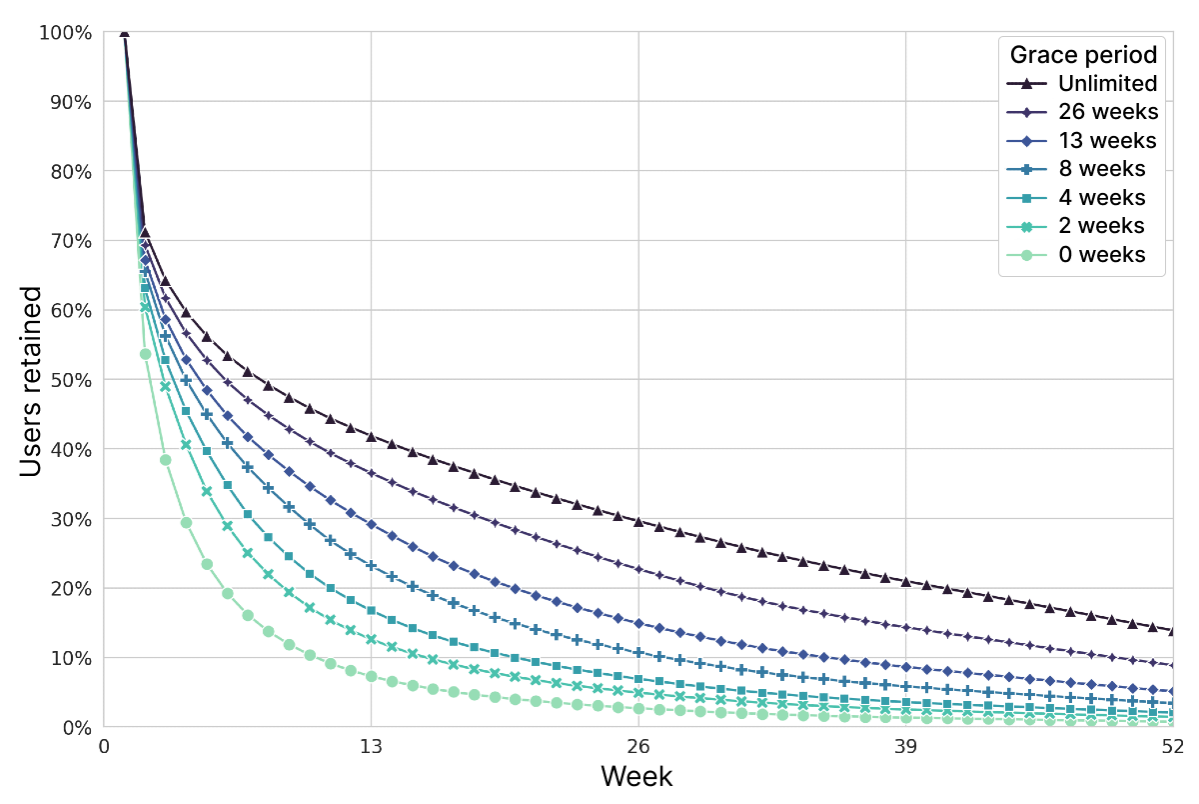
User retention, calculated by different grace periods of inactivity. In total, 792,692 users were tracked starting from their first recorded fast. Grace periods extended out to 104 weeks for the unlimited definition.

Figure 1 displays the retention patterns for the LIFE apps over the course of 52 weeks, calculated using 7 different fasting activity grace periods. There was an immediate drop of 28.7% of users (227,867/792,692) who never recorded a fast beyond week 1. Under the unlimited grace period, where up to 102 weeks of no fasting records were permitted, 41.9%, 29.6%, 21%, and 13.9% of users were retained at 13, 26, 39, and 52 weeks, respectively. At the other extreme, 0-week grace period retention (also known as full retention) captured a much smaller fraction of users (7.3%, 2.7%, 1.4%, and 0.8%, respectively).

Intermediate grace periods corresponded naturally to intermediate retention rates (Figure 1). For all remaining analyses in this study, we opted to use the 4-week grace period definition of retention because it allowed us to study the evident variability of use while precluding highly prolonged inactivity. These users recorded a fast approximately every month at a minimum. Retention rates under this definition were 16.7%, 6.9%, 3.6%, and 2.1% at 13, 26, 39, and 52 weeks, respectively. While users may have slowly increased their fasting frequency, taken a break, or ramped down at the end, exploring such behavioral dynamics falls outside the scope of this study.

### Demographics

**Figure 2:**
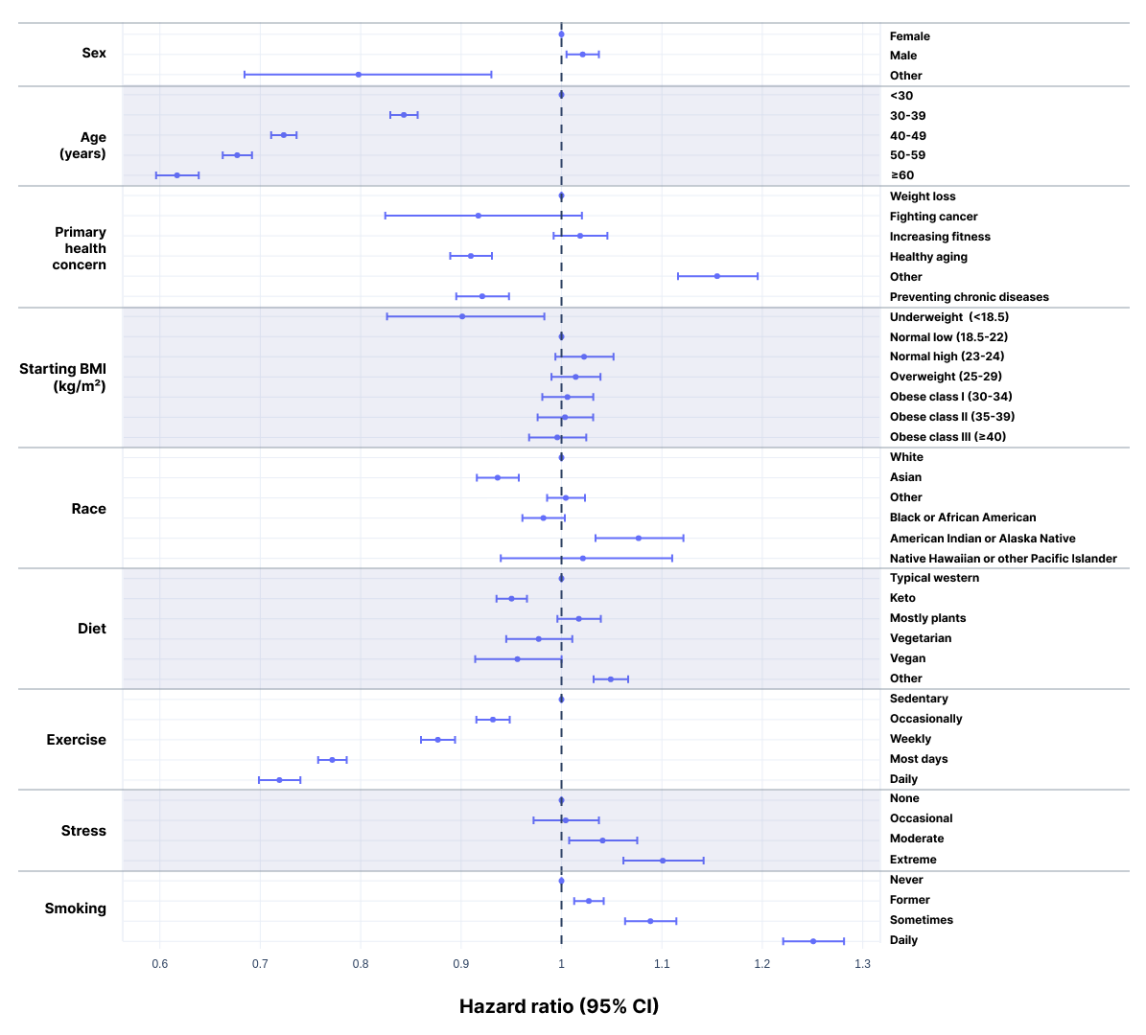
Hazard ratios with 95% CIs for failure to retain. Based on the Cox proportional hazards model over the 52-week study. HR=1 corresponds to the reference values: female, age <30 years, primary health concern as weight loss, starting BMI in the normal low category, white, typical western diet, sedentary, and never smoker. HR<1 reflects higher retention rates.

Retention using the 4-week grace period differed substantially by several demographic criteria (Multimedia Appendix 1). The Cox proportional hazards regression model built over the first 52 weeks confirmed that several factors were significant, even after controlling for other factors (Multimedia Appendix 1; Figure 2). While many demographic and behavioral factors were found to correlate with retention, 4 trends were particularly notable in the Cox model. Older users had higher retention—a hazard ratio of 0.617 (95% CI 0.596-0.639) for users aged ≥60 years means they are estimated to be about 38% less likely to drop each week than users <30 years. Similarly, increasing levels of exercise (as reported at baseline) reflected much greater retention, with daily exercisers dropping about 28% less often than users with a sedentary lifestyle. Conversely, stress and smoking conferred lower retention rates—10% and 25% higher drop rates respectively for users with extreme stress or daily smoking habits relative to users who have no stress and never smoked. While losing weight was the most common primary health concern, those users’ retention was substantially lower than for users whose primary concerns were healthy aging and preventing chronic disease. Sex and starting BMI appeared to have only small effects on retention.

### Fasting Practices and Patterns

#### Weekly Fasting Frequency

Even when retained, user fasting behavior is likely to change over time. We examined fasting patterns based mainly on the first 26 weeks among users retained that long. The 26-week period is long enough to see what long-term use of the fasting apps is like, while affording a larger sample size than looking only at users who were retained at 52 weeks. It also avoids overweighting the first few weeks of use when we had the largest sample but while users were still establishing their fasting routines.

**Figure 3:**
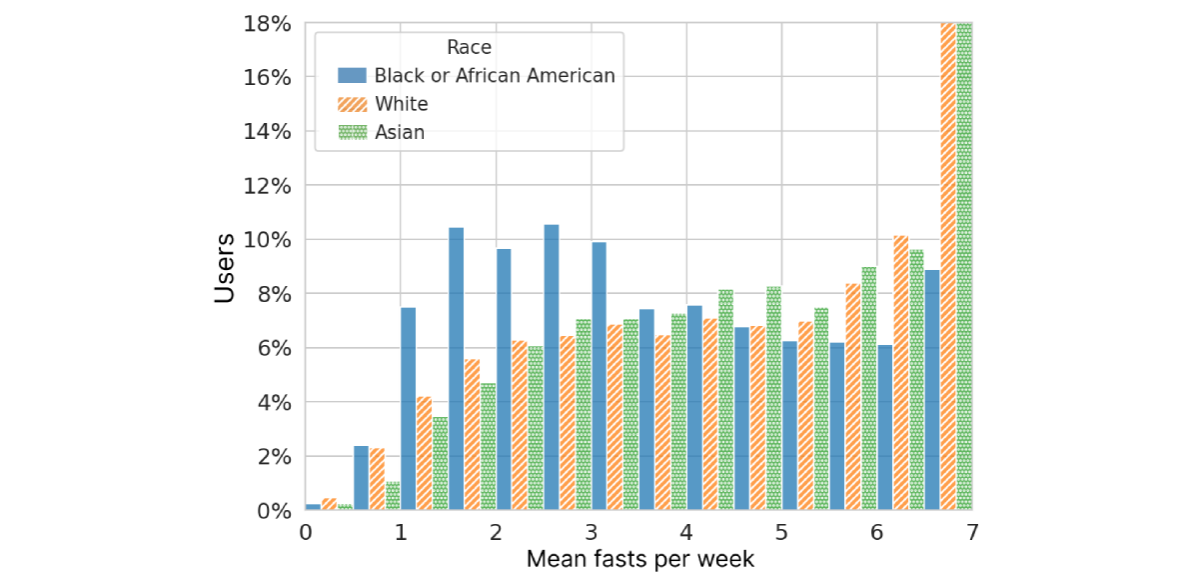
Fasting frequency statistics for users retained at 26 weeks, averaged over the first 26 weeks of use and grouped by self-reported race. Bins are half-fast width, left-inclusive, and include 7 in the highest bin.

The most common days to start a fast were Sunday, Monday, and Tuesday, whereas Friday and Saturday were the least popular. We also examined the distribution of fasts per user per week over the first 26 weeks for 54,811 users retained at 26 weeks using the 4-week grace period. The mean frequency was 4.25 (SD 1.91) fasts per week. Fasting frequency was approximately bimodal, with a broad peak centered on 3 fasts per week and a sharp peak at 7. Slightly more than one-quarter (13,981/54,881, 25.5%) of the users fasted 6 to 7 times per week. In Figure 3, weekly fasting frequency is shown separately for the 3 most common self-reported race values. The differences suggest large cultural influences on a user’s choice of fasting routine. Older users were also much more likely to fast 6 to 7 times per week than younger users (4033/11,768, 34.3%, vs 1521/9572, 15.9%) for users ≥50 years versus those <30 years.

#### Fasting Lengths

**Figure 4:**
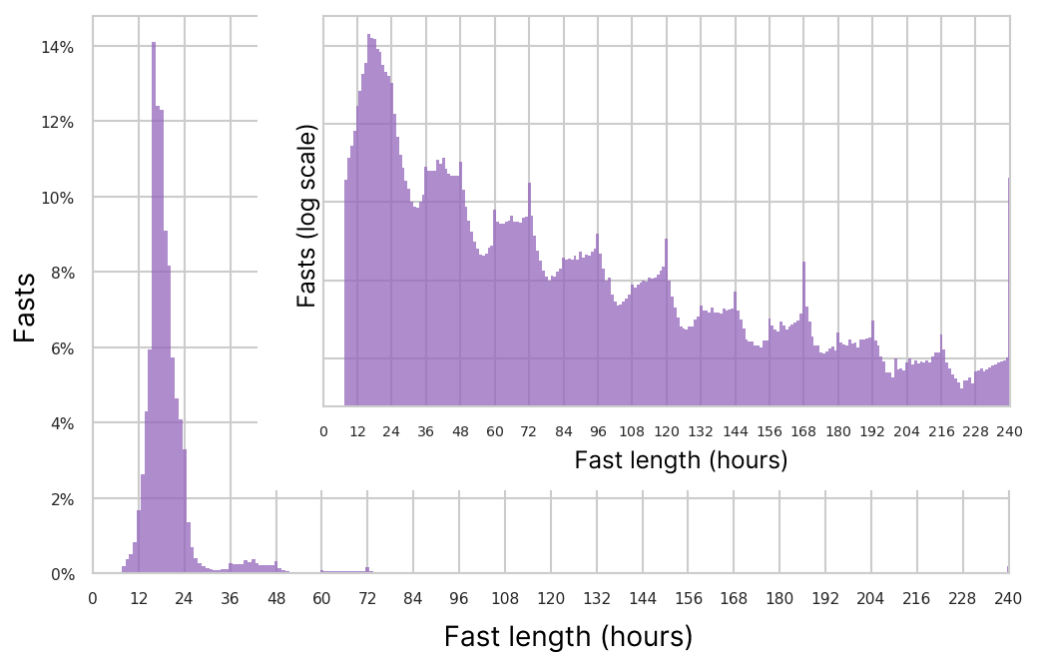
Histogram of fast lengths and a log scale histogram inset.

**Figure 5:**
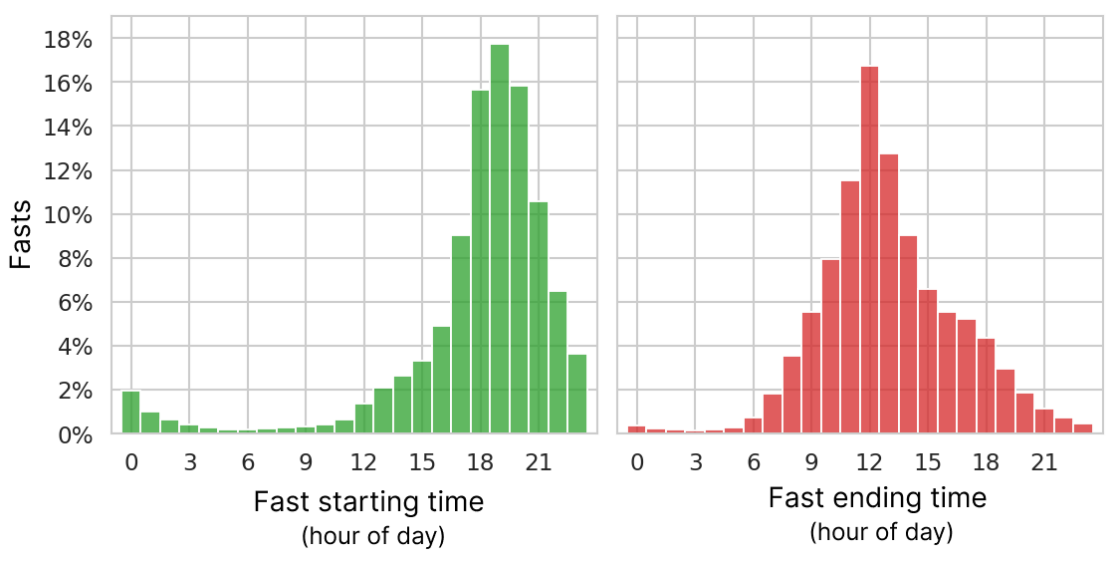
Distribution of starting and ending hour of fasts in local time.

The most common fasting length of the 26 million fasts analyzed over the entire length of the study was 16 hours. The mean and median lengths were 21.0 and 18.0 hours, respectively, while the lower and upper quartiles were 16.1 and 20.9 hours. Figure 4 shows the complete distribution of fasting lengths. The modal starting and ending hours were 7 PM and noon, respectively (Figure 5). A total of 93.5% (24,289,517/25,983,817) of fasts were ≤32 hours, typically spanning a single night. A pattern of multiday fasts is evident when plotted on the log scale in Figure 4 (inset), with smaller peaks for each additional day and clear spikes at precise multiples of 24 hours.

**Figure 6:**
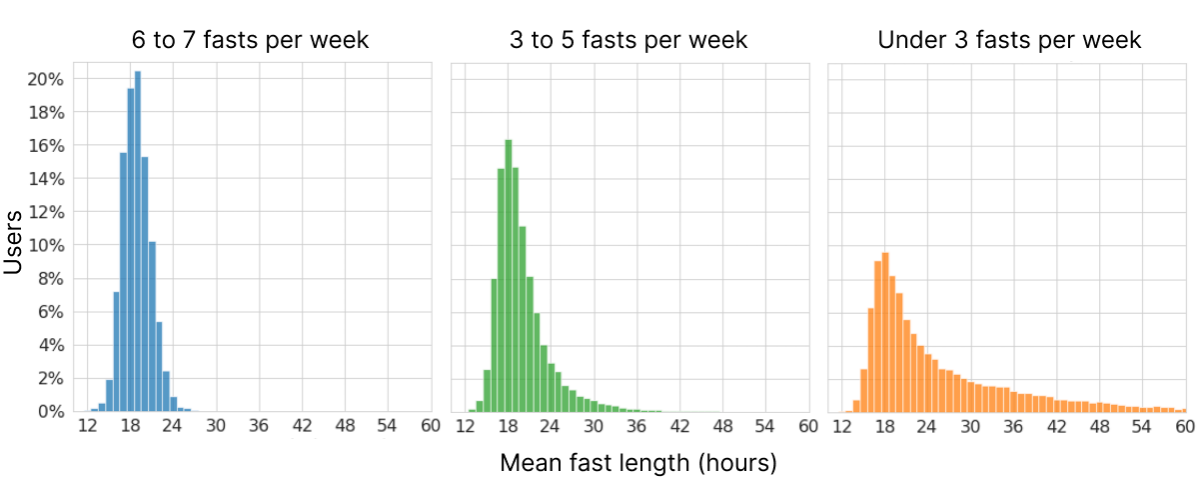
Distribution of average fast lengths per user across the first 26 weeks for users still retained at 26 weeks, broken down by weekly fasting frequency.

We also examined the average fast length by user for the 54,881 users who were retained at 26 weeks under the 4-week grace period definition. Figure 6 shows those 26-week averages broken down by user fasting frequency. Overall, 8.2% (4506/54,881) of users had a mean fast length of >32 hours, indicating a pattern of multiday fasts. As expected, among users who fasted <3 times per week, a much larger fraction (4055/17,057, 23.7%) was in the multiday zone of >32-hour average fasts, although the modal average was 18 hours. The average fast lengths for users who fasted 6 to 7 times per week also varied greatly, peaking at 19 hours.

Combining fasting length and frequency, the cumulative mean EFH per day was 5.0 at 26 weeks, which would correspond to a daily fasting routine of 17 hours.

### Weight Change

#### Demographics

We analyzed weight change for the 161,346 users who met the 4-week grace period retention criteria and recorded multiple weights in the fasting apps. From the univariate perspective, weight change as an outcome varied by several factors, including age, primary health concern, starting BMI, and EFH per day (Multimedia Appendix 2).

**Figure 7:**
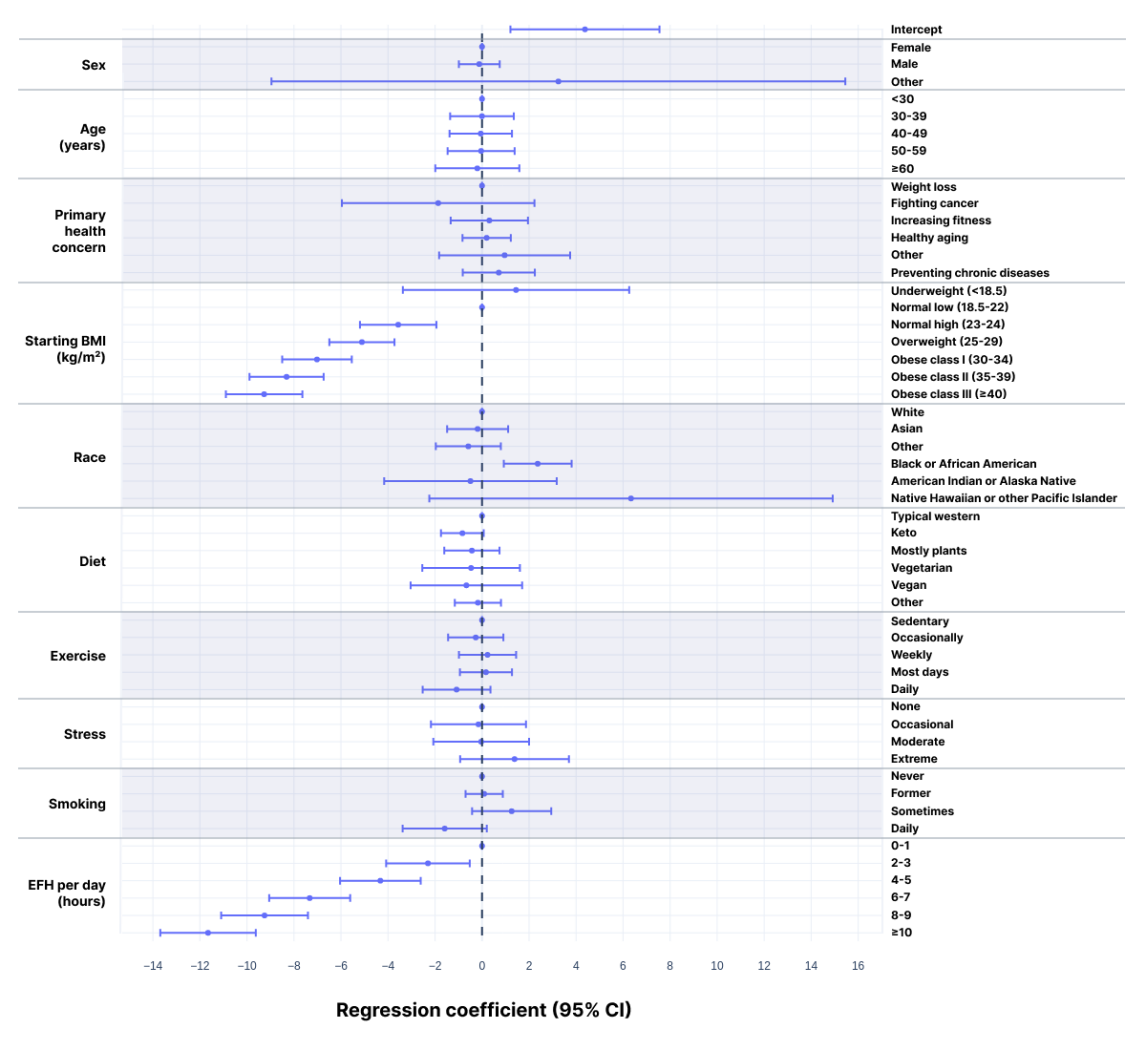
Regression coefficients with 95% CIs for weight change at 26 weeks. The model was built using ordinary least squares linear regression for the 1252 users who had answers for all variables and a weight recorded in week 26 (*R*^2^=0.334). Coefficients are shown as zero for the reference states: female, age <30 years, primary health concern as weight loss, starting BMI in the normal low category, white, typical western diet, sedentary, and never smoker. The regression coefficients reflect the difference in percent weight change at 26 weeks relative to the reference state for that category.

To address the correlation and confounding among variables, we conducted an ordinary least squares regression analysis of weight change at the 26-week time point. At 26 weeks, there were 1252 users with a recorded weight and values for all input variables. The result was that the only factors with *P*<.05 were starting BMI, EFH per day, and Black or African American race (Multimedia Appendix 2; Figure 7); *R*^2^=0.334. Results were similar for the models built at weeks 13, 39, and 52 (data not shown).

**Figure 8:**
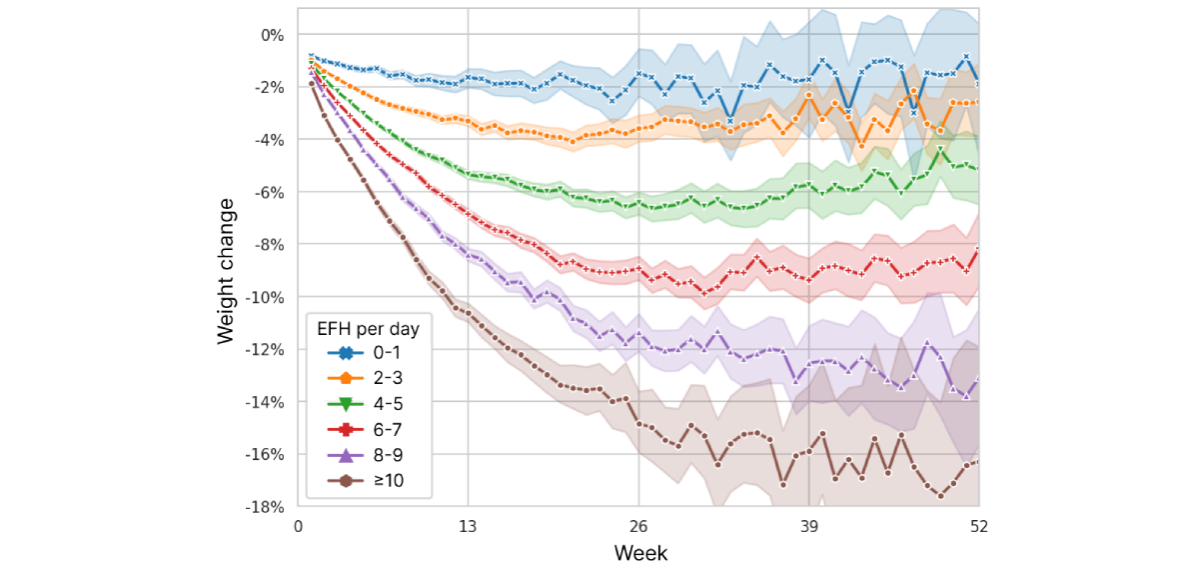
Weight change over time, stratified by users’ cumulative average extended fasting hours (EFH) per day. Excludes users with underweight starting BMI. Mean values are plotted with 95% CIs represented by shading.

**Figure 9:**
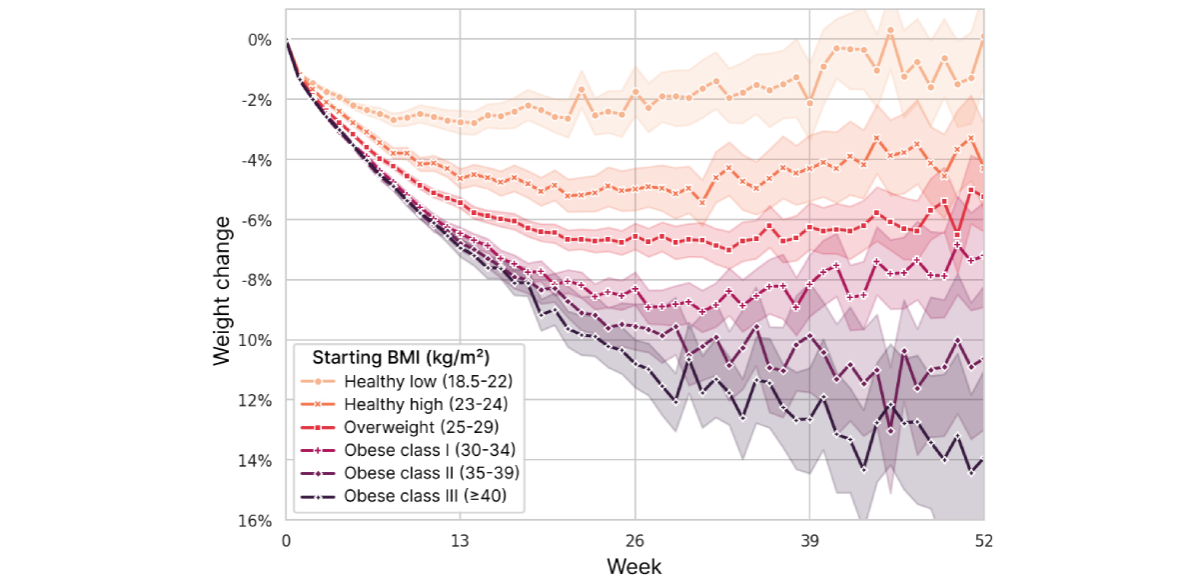
Weight change over time, stratified by user’s starting BMI category. Mean values are plotted with 95% CIs represented by shading.

We further graphically explored the 52-week patterns of weight change relative to EFH per day and starting BMI, which emerged as the main variables explaining variability in weight change. Figure 8 depicts weight change for users who are not categorized as underweight binned weekly based on their cumulative average EFH per day. While users fasting less than 2 EFH per day lost only about 2% of their starting weight by 26 weeks, users with more extensive fasting lost more than 1% of their starting weight for each additional hour of EFH per day. Within each EFH per day bin, weight change appeared to eventually plateau, with weight loss continuing longer at higher levels of fasting. Weight loss continued for 39 weeks for users with ≥8 EFH per day before plateauing. A graph of weight change stratified by starting BMI is shown in Figure 9.

**Figure 10:**
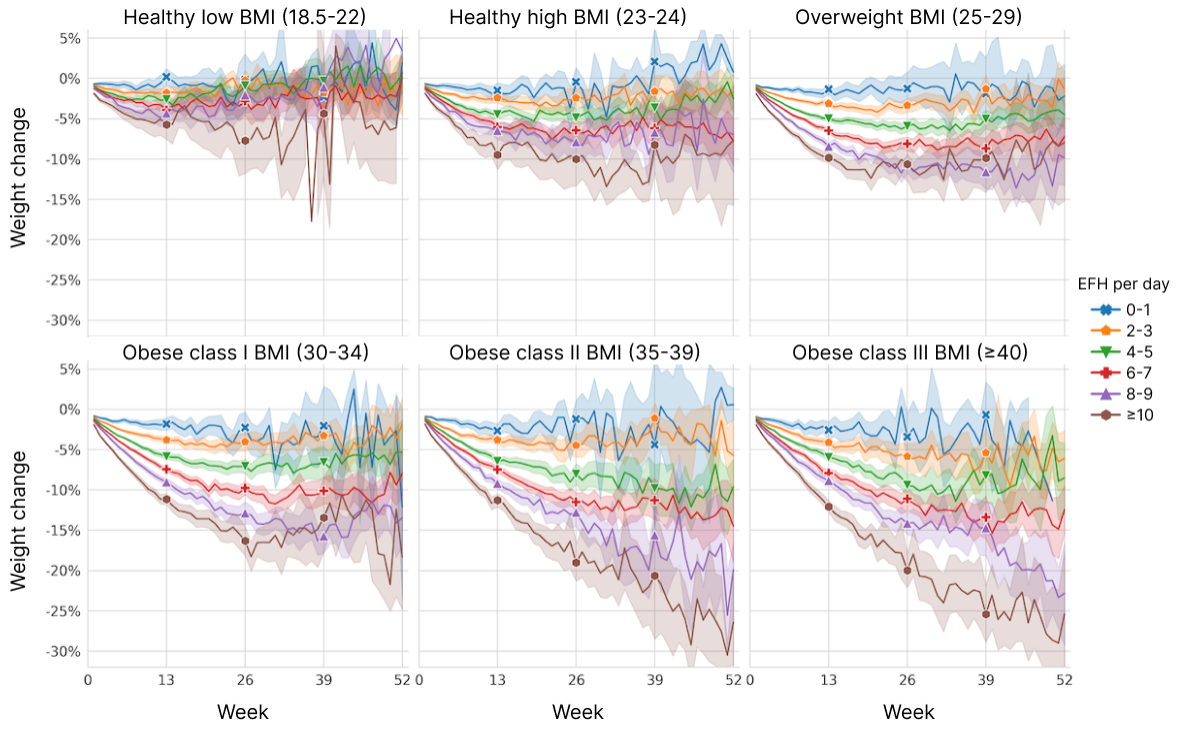
Weight change over time, stratified by user’s starting BMI level and cumulative average extended fasting hours (EFH) per day. Mean values are plotted with 95% CIs represented by shading.

We examined the combined effects of starting BMI and fasting quantity by plotting the EFH per day strata separately for each starting BMI category (Figure 10). Within each category, the effect of increasing EFH per day appears to be approximately linear, as seen previously in Figure 8, but the scale at which extended fasting impacts weight loss increases with higher BMI. Similarly, it is clear that the starting BMI is still predictive of weight loss, even after accounting for the amount of fasting. The evident interaction between these 2 factors was confirmed by rebuilding the 26-week regression model with the addition of an interaction term for continuous measures of starting BMI and EFH per day. In that analysis, the *P* value for the interaction term was <.001, whereas the *P* values for the EFH per day bins increased to >.05. *R*^2^ increased slightly to 0.356.

#### Weight Loss Thresholds

**Figure 11:**
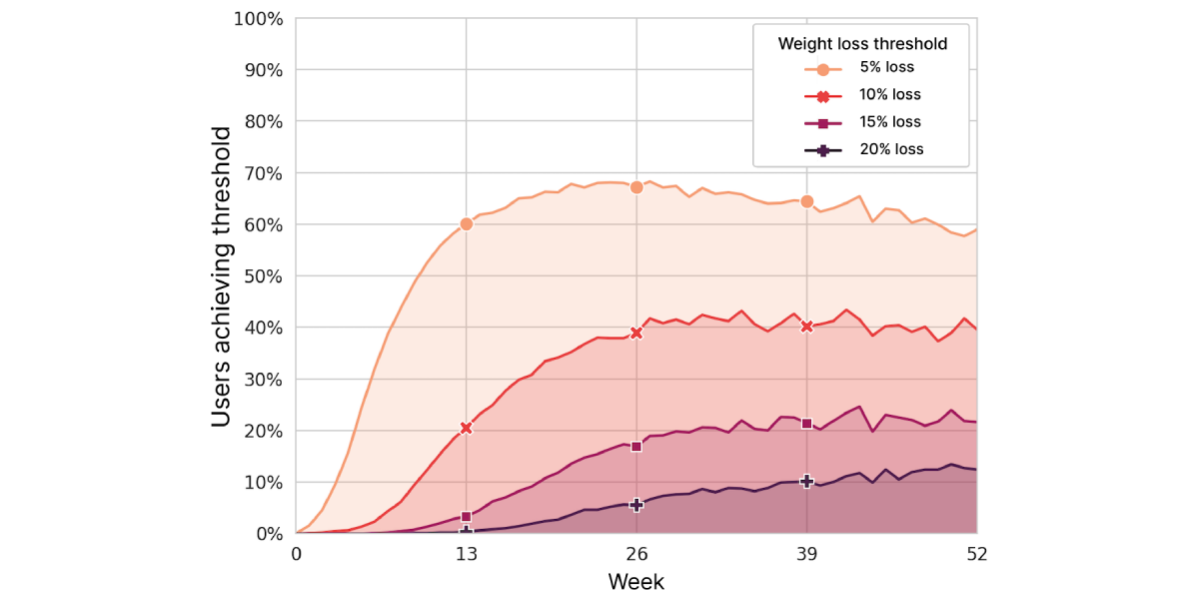
Percentage of users with obese or overweight starting BMI (≥25 kg/m^2^) who achieved 5%, 10%, 15%, and 20% weight loss by week.

We also examined the number of users who achieved certain thresholds of weight loss. Figure 11 shows the proportion of users with starting BMI ≥25 kg/m^2^ (ie, overweight or obese) who reached weight loss of 5%, 10%, 15%, and 20% over time. Success in reaching the 5% weight loss threshold was mostly achieved in the first 13 weeks and plateaued or peaked at 26 weeks. By 26 weeks, 67.2% (1475/2194) had lost at least 5% of their starting weight, and 38.9% (854/2194) had lost at least 10% of body weight. Reaching higher weight loss thresholds generally took much longer to achieve, with gradually larger fractions of users reaching them in 52 weeks.

## Discussion

### Context

This study is the largest examination of IF conducted to date and is orders of magnitude larger than any previous effort [5,7,12,31-33]. Owing to the use of mobile apps to record fasting events and weight, we were able to document real-world behavior and results, including both retention and weight change. Unlike most prior studies, we included people with healthy weight or underweight rather than just people categorized as overweight or obese, and our population covered extensive demographic variability, including an age range of 18 to 100 years.

### Retention

The spectrum of retention metrics (Figure 1) shows that there are many users who consistently used the apps to record fasts every week for months (0-week grace period). Other users took breaks lasting weeks or months, but came back to the apps later—13.9% of users recorded a fast during weeks 52 to 104 (ie, retention at week 52 with an unlimited grace period), which is an underestimate because most users downloaded the app less than 2 years before the end of the study. Whether users are engaged in IF during reporting gaps is unknown, but these variable use patterns are likely typical for mobile health apps, as well as health behavior in general [34,35].

Retention statistics for mobile apps are not commonly available for proprietary apps. An analysis by AppsFlyer found that day 30 retention (fraction of original users active on day 30) for health and fitness apps in the United States in 2020 averaged <6% [36]. The week-12 retention statistics (fraction of original users active during week 12) for all apps in 2020 were 3.6% for Android and 5.1% for iOS. This 12-week retention definition is more generous than our 0-week grace period definition, yet the retention we observed at that time point (Figure 1) was greater, perhaps owing to the simple core utility of the fasting app for tracking the timing of fasts.

Age was the best predictor of retention in our study, which is consistent with other analyses of retention predictors for lifestyle interventions [37]. Older users may become more fixed in new habits, appreciate the consistency of using apps regularly, and be less likely to try out multiple competing apps. They are also more likely to have serious health concerns, such as healthy aging, which may increase their motivation to adhere to new interventions. Users whose stated primary health concern was weight loss were younger and had the lowest retention rate.

Several other variables were notable in their relationship to retention in both univariate and multivariate analyses (Multimedia Appendix 1). Consistent with previous reports [37,38], 26-week retention was more than twice as high for daily exercisers versus sedentary users. This was also concordant with previous findings of higher dietary compliance among those who exercise regularly [39]. Stress has been shown to predict poor adherence to weight loss programs [37], and users of the LIFE apps reporting extreme stress had the lowest retention. Smoking conferred lower retention rates compared with nonsmoking, which is also consistent with previous reports [40,41]. Interestingly, retention differences by sex, diet, and starting BMI were among the smallest.

### Fasting Patterns and Weight Change

The real-world spectrum of fasting behavior documented in our study shows variable and flexible adherence to IF regimens, making specific idealized fasting protocols hard to discern in the data. We did clearly see a group of 25.5% of users who practice a daily, or nearly daily, fasting routine (≥6 days per week) averaged over the first 26 weeks, whereas the rest skip multiple days per week. Among users who fast, on average, fewer than 3 times per week, the majority fast under 24 hours, suggesting that they are more sporadic in their fasting or less vigorous in tracking in the apps. A total of 7.5% of users had average fasts over 32 hours, likely corresponding to the extended paradigms of IF such as 5:2 and alternate day fasting.

Owing to the multidimensional gradations of fasting patterns, we proposed the concept of EFH per day, as a metric to quantify fasting across all users. EFH per day combines fasting frequency and fasting length into a single measure and serves to unify the various fasting regimens for analysis. EFH per day was predictive of weight loss in a nearly linear fashion (Multimedia Appendix 2; Figures 8 and 10), supporting it as a relevant framework for quantifying fasting. We also showed that the magnitude of the fasting effect varied by starting BMI, with greater weight loss in individuals with higher levels of obesity practicing the same level of IF. Our findings have clear implications for people who wish to lose weight by using IF. The daily 16:8 IF routine that is commonly promoted is a minimum for those who wish to lose more than a few percent of their weight.

To explain these results, we hypothesize that the correlation between EFH per day and weight loss and the interaction with starting BMI can be attributed primarily to differences in caloric restriction. In previous studies, those who practiced alternate day fasting, the 5:2 diet, or time-restricted eating reduced their daily calorie intake by 10% to 30% [42]. With shorter eating windows, users with low starting BMI may be able to consume sufficient calories to maintain their weight, while users with higher BMI cannot, resulting in disproportionate weight loss. Various IF regimens have been shown to be as effective for weight loss as intentional caloric restriction [6,8,43,44], although IF might be easier to adopt and follow in the long term [13,45].

The weight loss effects of longer fasts may additionally be driven by the metabolic switch from glucose to ketones derived from fat tissue and free fatty acids [46]. This switch has previously been associated with weight loss [20] and occurs between 12 hours and 24 hours into a fast, depending on previous carbohydrate intake and energy expenditure [46,47]. A primary benefit of the switch from use of glucose to free fatty acids and ketones is the mobilization of body fat stores while preserving muscle mass, thereby improving body composition. Associated improvements in insulin sensitivity, visceral fat mass, and systemic inflammation may persist at the end of each fast when the system reverts to glucose metabolism, in part because of preserved muscle mass [19]. These effects are amplified in longer fasts, as insulin sensitivity reaches a nadir at 54 hours.

Analysis of weight loss threshold achievement facilitates the comparison of IF with other interventions. By 26 weeks, 67.2% (1475/2194) of users with overweight and obese starting BMI lost at least 5% of their starting weight. This is comparable to the results achieved by users of the Diabetes Prevention Program through the Noom platform, 64% of whom lost over 5% of their body weight in 24 weeks [48]. In our study, 38.9% (854/2194) of users with BMI ≥25 kg/m^2^ lost at least 10% of their body weight in 26 weeks, while this threshold was achieved by only 23% of Noom users in the same amount of time [49]. In another comparison, only 25% of the participants enrolled in the Livongo Diabetes Prevention Program lost more than 10% of their body weight by 54 weeks, with most users achieving 5% weight loss [50].

While it is common for people who lose weight to be subject to weight regain [51], overweight users sustained most of their weight loss at 52 weeks, with users in obese class II and III (BMI ≥35 kg/m^2^) trending toward even more weight loss at 52 weeks. In contrast, users whose starting BMI classified them as underweight had no weight change on average at 13 weeks and proceeded to gain weight if they continued with the apps out to 52 weeks. This is an important finding because of the frequently raised concern that IF may promote eating disorders [52]. Our findings suggest that IF is generally a safe practice even for users at the low end of BMI because even those users who fasted extensively tended to lose little weight (Figure 10).

Age was positively associated with greater weight loss, consistent with previous findings [53]. The effect we observed is explained by more fasting by older users rather than as a consequence of metabolic or dietary differences, according to our multivariate model. This is consistent with a 2013 study that showed that, compared with younger participants, older adults lost significantly more weight after a diet and exercise intervention and were more successful at maintaining weight loss even after 3 years of an internet-based maintenance protocol [54]. The lack of weight loss difference between women and men is also consistent with previous findings [55].

### Conclusions

As of 2016, close to 50% of adults in the United States had tried to lose weight within the preceding 12 months according to Centers for Disease Control and Prevention data [56]. Moreover, as of 2018, over 40% of adults in the United States were considered obese [57]. In our study of mobile app users in the real-world setting, we found that IF is an effective strategy for weight loss for many people. Studies in people with obesity demonstrate that losing 10% of your body weight is enough to improve blood pressure and normalize cholesterol blood levels, while losing just 5% is enough to improve glycemic control, which is central to the prevention and management of diabetes [58]. In just 13 weeks, among LIFE apps users with a starting classification of overweight or obese, 60% of them lost 5% or more of their starting weight and 21% lost ≥10%, reflecting the potential clinical value that is achievable. These rates of success were higher in our users than in users of paid apps with active coaching, such as Noom and Livongo.

Self-reported data in this mobile setting offered many intriguing correlates of retention and weight loss. Meaningful factors included diet, exercise, stress, and smoking, all of which lend themselves well to mobile tracking, including integrated wearables, for reliable measurement and analysis.

### Future Work

To better understand the mechanisms and residual variability of weight loss by app users, we would like to study the caloric input and expenditure of users directly. This can be achieved by asking users to record their daily dietary intake and exercise. Tracking exercise is amenable to passive tracking with wearable technology, and many users of the LIFE apps (specifically LX) already have regular data ingestion established with the most common fitness trackers. Such an additional study could help resolve the somewhat surprising finding that the diet and exercise habits self-reported at the beginning of the study did not correlate with weight change.

Although weight management is a clinically important objective, other clinically relevant outcomes could be measured and correlated with fasting behavior. These include assessments of mental and physical health, disease incidence, insulin resistance, medical costs, and professional and educational absenteeism. Advocates of IF point to studies in animal models and humans that suggest many of these benefits [11,14,59-61]. Facilitated by mobile and digital technology, we may be able to evaluate real-world evidence for these promises and tease apart their etiology.

Finally, studies show that social support improves health and well-being, and that people who have strong support networks are more likely to lose weight than those who do not [62,63]. The LIFE apps have a social “Circles” feature, where users can communicate with other users within the app. An analysis of circle participation is a subject for future work, but preliminary results suggest that retention is higher for users who are socially active in the app.

### Limitations

The primary limitation of this study was that most data were self-reported, except for some weight values that were entered by smart scales. This limitation is compensated for by the large sample size of the study.

The observed weight change averages may be potentially confounded by users who stopped recording weights or even stopped using the app because of a lack of progress. Conversely, users who achieved success might have been less motivated to continue recording fasts and weights. Similarly, users may have been more likely to weigh themselves and record their weight in the app if they had lost weight, which could then exaggerate the weight loss estimates in this study. These effects may be challenging to untangle, but the trends and correlates of weight change identified should be robust.

Owing to the limited observational nature of this study, users who fasted longer may have adopted other diet-related practices more than users who fasted less without our knowledge. Similarly, we did not have information about users’ previous experience with IF. Given that the largest retention losses and greatest rates of weight change occurred in the earliest weeks of app use, previous fasting experience or even recent weight changes could skew the reported progress. In an IF-naive population, we expect weight loss to be relatively larger.

Another limitation of the study is that, due to being observational, it lacked explicit controls. However, this limitation was offset by the wide range of fasting behaviors among users. We used this natural variability as a form of self-directed intervention, which allowed us to contrast and quantify the effects of different levels of fasting on a much broader scale than would be feasible for a randomized controlled trial.

## Appendix

Multimedia Appendix 1 Baseline demographics and retention at 13-week intervals using the 4-week grace period retention definition. Hazard ratios and corresponding *P* values are based on the 52-week Cox proportional hazards regression model applied to retention. Hazard ratios <1.0 reflect greater rates of retention.

Multimedia Appendix 2 Weight change at weeks 13, 26, 39, and 52 using the 4-week grace period retention definition relative to user demographics. Starting BMI (mean, SD) for each demographic category is included for context. Sample sizes reflect the maximum eligible users at each time point. Regression coefficients and *P* values refer to an ordinary least squares model of weight change at 26 weeks (n=1252). The intercept in the model was 4.378 (*P*=.007). Baseline BMI values for the EFH per day groupings are based on the 26-week cohort used in the regression analysis.

## Abbreviations

EFH: extended fasting hours
IF: intermittent fasting
LFT: LIFE Fasting Tracker
LX: LIFE Extend
