# Supplementary material for: Retention, Fasting Patterns, and Weight Loss With an Intermittent Fasting App: Large-Scale, 52-Week Observational Study
Source: JMIR Mhealth Uhealth. 2022 Oct 4;10(10):e35896. doi: 10.2196/35896 (PMC9579929; doi:10.2196/35896)
Supplement: Multimedia Appendix 1 [file mhealth_v10i10e35896_app1.pdf]

Multimedia Appendix 1. This is a Multimedia Appendix to a full manuscript by Torres et al. published 2022 in the J Med Internet Res mHealth and uHealth.

Demographic characteristic	Users, n (% of column)	Retention, n (% of row)				26-week regression	
	0 weeks	13 weeks	26 weeks	39 weeks	52 weeks	Hazard ratio	*P* value
**Sex**							
Female	644,378 (81.3)	108,590 (16.9)	44,927 (7)	23,150 (3.6)	13,385 (2.1)	1.000	N/A[a]
Male	146,961 (18.5)	23,946 (16.3)	9869 (6.7)	5236 (3.6)	3066 (2.1)	1.021	.01
Other	1353 (0.2)	239 (17.7)	85 (6.3)	44 (3.3)	27 (2)	0.800	.004
**Age (years)**							
<30	241,531 (30.5)	27,411 (11.3)	9572 (4)	4392 (1.8)	2326 (1)	1.000	N/A
30-39	270,011 (34.1)	42,717 (15.8)	16,776 (6.2)	8393 (3.1)	4779 (1.8)	0.843	<.001
40-49	182,411 (23)	37,971 (20.8)	16,765 (9.2)	9078 (5)	5448 (3)	0.723	<.001
50-59	77,346 (9.8)	18,685 (24.2)	8713 (11.3)	4823 (6.2)	2828 (3.7)	0.677	<.001
≥60	21,393 (2.7)	5991 (28)	3055 (14.3)	1744 (8.2)	1097 (5.1)	0.617	<.001
**Primary health concern**							
Losing weight	456,287 (77.3)	76,874 (16.8)	31,276 (6.9)	15,562 (3.4)	8559 (1.9)	1.000	N/A
Healthy aging	46,571 (7.9)	11,519 (24.7)	5717 (12.3)	3403 (7.3)	2202 (4.7)	0.910	<.001
Increasing fitness	34,874 (5.9)	5960 (17.1)	2592 (7.4)	1348 (3.9)	774 (2.2)	1.019	.17
Preventing chronic diseases	27,955 (4.7)	6138 (22)	2753 (9.8)	1546 (5.5)	920 (3.3)	0.921	<.001
Fighting cancer	2041 (0.3)	419 (20.5)	171 (8.4)	104 (5.1)	59 (2.9)	0.917	.11
Other	22,220 (3.8)	3365 (15.1)	1302 (5.9)	627 (2.8)	342 (1.5)	1.155	<.001

	0 weeks	13 weeks	26 weeks	39 weeks	52 weeks	Hazard ratio	*P* value
**Starting BMI (kg/m^2)**							
Underweight (<18.5)	2124 (0.6)	327 (15.4)	141 (6.6)	72 (3.4)	50 (2.4)	0.901	.02
Healthy low (18.5-22)	34,037 (9)	6105 (17.9)	2508 (7.4)	1267 (3.7)	784 (2.3)	1.000	N/A
Healthy high (23-24)	37,671 (10)	6572 (17.4)	2820 (7.5)	1500 (4)	890 (2.4)	1.023	.12
Overweight (25-29)	115,281 (30.5)	20,366 (17.7)	8382 (7.3)	4310 (3.7)	2587 (2.2)	1.014	.25
Obese class I (30-34)	92,714 (24.5)	15,985 (17.2)	6444 (7)	3202 (3.5)	1851 (2)	1.006	.65
Obese class II (35-39)	52,441 (13.9)	8783 (16.7)	3401 (6.5)	1656 (3.2)	911 (1.7)	1.0035	.81
Obese class III (≥40)	43,529 (11.5)	6955 (16)	2711 (6.2)	1307 (3)	701 (1.6)	0.996	.77
**Race**							
White	221,959 (59.5)	40,588 (18.3)	17,731 (8)	9342 (4.2)	5350 (2.4)	1.000	N/A
Asian	42,108 (11.3)	6919 (16.4)	2827 (6.7)	1383 (3.3)	812 (1.9)	0.936	<.001
Black or African American	38,488 (10.3)	6850 (17.8)	2828 (7.3)	1455 (3.8)	840 (2.2)	0.982	.1
American Indian or Alaska Native	10,590 (2.8)	1483 (14)	564 (5.3)	260 (2.5)	126 (1.2)	1.077	<.001
Native Hawaiian or other Pacific Islander	2129 (0.6)	349 (16.4)	141 (6.6)	71 (3.3)	38 (1.8)	1.021	.62
Other race	57,596 (15.4)	9195 (16)	3546 (6.2)	1656 (2.9)	909 (1.6)	1.004	.65
**Diet**							
Typical western	82,677 (34.5)	16,262 (19.7)	7418 (9)	3959 (4.8)	2265 (2.7)	1.000	N/A
Keto	62,754 (26.2)	13,920 (22.2)	5974 (9.5)	3111 (5)	1777 (2.8)	0.950	<.001
Mostly plants	24,778 (10.3)	5280 (21.3)	2374 (9.6)	1284 (5.2)	776 (3.1)	1.017	.12
Vegetarian	8874 (3.7)	1894 (21.3)	848 (9.6)	447 (5)	274 (3.1)	0.977	.18
Vegan	5020 (2.1)	1121 (22.3)	10.8 (544)	5.8 (292)	3.5 (178)	0.956	.05
Other	55,767 (23.2)	10,150 (18.2)	4535 (8.1)	2475 (4.4)	1431 (2.6)	1.049	<.001

	0 weeks	13 weeks	26 weeks	39 weeks	52 weeks	Hazard ratio	*P* value
**Exercise**							
Sedentary	51,214 (19.7)	7827 (15.3)	3088 (6)	1596 (3.1)	856 (1.7)	1.000	N/A
Occasionally	67,275 (25.9)	11,862 (17.6)	5058 (7.5)	2606 (3.9)	1458 (2.2)	0.932	<.001
Weekly	48,914 (18.8)	9711 (19.9)	4255 (8.7)	2249 (4.6)	1342 (2.7)	0.877	<.001
Most days	75,333 (29.0)	18,754 (24.9)	8894 (11.8)	883 (6.5)	2890 (3.8)	0.772	<.001
Daily	17,267 (6.6)	4527 (26.2)	2274 (13.2)	1248 (7.2)	750 (4.3)	0.719	<.001
**Stress**							
None	15,309 (4.5)	3063 (20)	1329 (8.7)	645 (4.2)	385 (2.5)	1.000	N/A
Occasional	133,222 (39.3)	26,234 (19.7)	8.7 (11,595)	4.5 (6008)	2.6 (3456)	1.004	.80
Moderate	151,094 (44.6)	25,835 (17.1)	10,719 (7.1)	5495 (3.6)	3110 (2.1)	1.041	.02
Extreme	39,336 (11.6)	5928 (15.1)	2325 (5.9)	1157 (2.9)	648 (1.6)	1.101	<.001
**Smoking**							
Never	195,039 (61.2)	38,541 (19.8)	17,047 (8.7)	8939 (4.6)	5177 (2.7)	1.000	N/A
Former	75,749 (23.8)	15,317 (20.2)	6619 (8.7)	3564 (4.7)	2059 (2.7)	1.027	<.001
Sometimes	24,284 (7.6)	3497 (14.4)	5.6 (1364)	656 (2.7)	346 (1.4)	1.089	<.001
Daily	23,392 (7.3)	2732 (11.7)	1017 (4.3)	467 (2)	250 (1.1)	1.251	<.001

[a]N/A: not applicable.
