# Supplementary material for: Retention, Fasting Patterns, and Weight Loss With an Intermittent Fasting App: Large-Scale, 52-Week Observational Study
Source: JMIR Mhealth Uhealth. 2022 Oct 4;10(10):e35896. doi: 10.2196/35896 (PMC9579929; doi:10.2196/35896)
Supplement: Multimedia Appendix 2 [file mhealth_v10i10e35896_app2.pdf]

Multimedia Appendix 2. This is a Multimedia Appendix to a full manuscript by Torres et al. published 2022 in the J Med Internet Res mHealth and uHealth.

Demographic characteristic	Starting BMI (kg/m^2), SD	Change from starting BMI (%), SD				26-week regression	
	0 weeks (n=161,346)	13 weeks (n=8371)	26 weeks (n=2652)	39 weeks (n=1083)	52 weeks (n=577)	Coefficient	*P* value
**Sex**							
Female	31.5 (7.4)	−5.7 (4.7)	−7.5 (7.1)	−7.5 (8.6)	−6.9 (9.8)	0	N/A[a]
Male	32.0 (6.6)	−6.3 (5.2)	−7.1 (7.9)	−6.9 (9.7)	−6.3 (10.1)	−0.119	.79
Other	29.6 (8.0)	−6.8 (5.1)	−3.1 (3.5)	−3.6 (N/A[a])	N/A	3.247	.60
**Age (years)**							
<30	30.5 (7.4)	−5.3 (5.6)	−6.1 (8.1)	−4.6 (8.9)	−4.0 (9.6)	0	N/A
30-39	32.1 (7.4)	−5.8 (4.9)	−7.0 (7.5)	−7.4 (9.5)	−6.2 (10.3)	−0.003	0.99
40-49	31.9 (7.2)	−5.9 (4.6)	−7.8 (7.1)	−7.7 (8.9)	−7.0 (9.5)	−0.055	.94
50-59	31.8 (6.7)	−6.0 (4.5)	−8.0 (6.6)	−7.9 (8.0)	−7.8 (10.2)	−0.039	.96
≥60	31.2 (6.4)	−5.9 (4.1)	−8.2 (6.2)	−8.5 (7.4)	−8.5 (8.2)	−0.201	.83
**Primary health concern**							
Losing weight	32.4 (7.3)	−6.1 (4.8)	−7.8 (7.3)	−7.9 (9.2)	−7.1 (10.1)	0	N/A
Healthy aging	27.7 (5.6)	−5.0 (4.6)	−6.3 (6.5)	−6.3 (7.0)	−5.8 (8.9)	0.196	.71
Increasing fitness	27.9 (6.0)	−4.8 (5.1)	−6.1 (7.4)	−5.3 (9.1)	−6.9 (10.7)	0.310	.71
Preventing chronic diseases	31.2 (7.3)	−5.8 (4.6)	−7.5 (6.5)	−7.2 (8.0)	−6.0 (6.6)	0.713	.36
Fighting cancer	29.6 (6.2)	−4.8 (4.0)	−5.6 (6.8)	−9.2 (9.0)	−7.8 (N/A)	−1.865	.37
Other	29.6 (7.0)	−5.4 (5.2)	−5.9 (8.1)	−5.0 (6.0)	−0.7 (8.5)	0.961	.50

	0 weeks (n=161,346)	13 weeks (n=8371)	26 weeks (n=2652)	39 weeks (n=1083)	52 weeks (n=577)	Coefficient	*P* value
**Starting BMI (kg/m2)**							
Underweight (<18.5)	17.4 (1.4)	0 (5.0)	0.6 (4.7)	0.9 (5.5)	4.9 (2.2)	1.446	.56
Healthy low (18.5-22)	21.5 (1.1)	−2.8 (4.5)	−1.7 (5.6)	−2.1 (5.6)	0.1 (5.5)	0	N/A
Healthy high (23-24)	24.1 (0.6)	−4.6 (4.6)	−5.0 (5.6)	−4.3 (6.2)	−4.3 (6.2)	−3.566	<.001
Overweight (25-29)	27.6 (1.4)	−5.4 (4.7)	−6.6 (6.4)	−6.3 (7.8)	−5.2 (8.4)	−5.110	<.001
Obese class I (30-34)	32.3 (1.4)	−6.5 (4.8)	−8.3 (7.1)	−8.2 (8.6)	−7.2 (10.1)	−7.021	<.001
Obese class II (35-39)	37.2 (1.4)	−6.8 (4.6)	−9.5 (7.8)	−9.9 (9.9)	−10.6 (11.0)	−8.315	<.001
Obese class III (≥40)	45.7 (5.6)	−6.9 (4.6)	−10.8 (7.6)	−12.6 (10.7)	−13.9 (11.8)	−9.269	<.001
**Race**							
White	31.6 (7.2)	−5.9 (4.8)	−7.7 (7.3)	−8.2 (9.3)	−7.4 (10.3)	0	N/A
Asian	28.5 (5.7)	−6.2 (5.1)	−7.0 (7.6)	−5.3 (7.5)	−5.5 (8.7)	−0.187	.78
Black or African American	35.3 (8.0)	−5.4 (4.9)	−6.1 (7.5)	−5.4 (7.1)	−7.2 (8.7)	2.368	.001
American Indian or Alaska Native	31.6 (7.1)	−6.4 (5.0)	−9.0 (5.6)	−12.8 (3.6)	−5.9 (15.4)	−0.493	.79
Native Hawaiian or other Pacific Islander	34.4 (8.3)	−5.4 (4.8)	−6.1 (0.5)	−15.8 (5.3)	3.8 (N/A)	6.337	.15
Other race	31.9 (7.1)	−6.5 (4.8)	−7.3 (7.5)	−5.7 (8.5)	−7.2 (9.5)	−0.584	.41
**Diet**							
Typical western	32.3 (7.3)	−5.8 (4.5)	−7.6 (6.4)	−7.8 (7.8)	−8.4 (9.8)	0	N/A
Keto	32.5 (7.5)	−6.4 (5.2)	−8.2 (8.4)	−7.7 (9.9)	−7.4 (11.1)	−0.835	.07
Mostly plants	29.5 (6.7)	−5.8 (4.9)	−6.5 (7.5)	−5.6 (9.1)	−5.4 (6.3)	−0.432	.47
Vegetarian	28.8 (6.9)	−5.2 (4.9)	−4.4 (7.0)	−7.7 (8.5)	−4.8 (9.1)	−0.466	.66
Vegan	28.4 (7.1)	−4.3 (5.6)	−7.0 (7.0)	−7.6 (6.4)	−3.0 (6.0)	−0.666	.58
Other	31.8 (7.4)	−5.9 (5.0)	−7.0 (8.2)	−8.1 (11.1)	−7.1 (11.8)	−0.176	.73

	0 weeks (n=161,346)	13 weeks (n=8371)	26 weeks (n=2652)	39 weeks (n=1083)	52 weeks (n=577)	Coefficient	*P* value
**Exercise**							
Sedentary	34.6 (8.5)	−6.2 (4.9)	−8.4 (7.1)	−8.9 (8.9)	−7.9 (9.6)	0	N/A
Occasionally	32.7 (7.2)	−5.8 (4.7)	−7.7 (7.4)	−8.1 (9.7)	−6.6 (11.7)	−0.268	.66
Weekly	31.2 (6.9)	−6.0 (4.5)	−7.1 (7.1)	−8.1 (8.7)	−8.0 (8.7)	0.234	.71
Most days	29.8 (6.3)	−5.8 (4.9)	−6.8 (7.4)	−6.6 (9.1)	−6.6 (9.9)	0.166	.77
Daily	28.9 (6.4)	−5.8 (5.1)	−8.3 (7.5)	−7.5 (8.8)	−7.7 (10.2)	−1.084	.14
**Stress**							
None	30.1 (6.6)	−6.0 (4.8)	−7.1 (7.2)	−7.9 (9.3)	−7.3 (11.8)	0	N/A
Occasional	31.3 (7.2)	−5.9 (4.7)	−7.6 (7.0)	−7.6 (8.5)	−6.8 (9.6)	−0.152	.88
Moderate	31.9 (7.4)	−5.8 (4.9)	−7.4 (7.4)	−7.6 (9.1)	−7.2 (9.8)	−0.036	.97
Extreme	32.8 (7.9)	−5.8 (5.2)	−6.5 (8.4)	−6.4 (10.1)	−7.5 (11.6)	1.383	.24
**Smoking**							
Never	31.7 (7.3)	−5.8 (4.7)	−7.6 (7.1)	−7.8 (8.9)	−6.9 (10.1)	0	N/A
Former	32.2 (7.4)	−6.1 (4.9)	−7.7 (7.1)	−7.5 (9.0)	−7.9 (10.2)	0.09	.82
Sometimes	31.2 (7.4)	−4.8 (5.4)	−4.5 (7.3)	−3.5 (8.8)	−2.5 (5.8)	1.26	.14
Daily	31.9 (7.5)	−7.1 (5.5)	−9.0 (9.1)	−8.8 (8.4)	−8.1 (7.6)	−1.588	.08
**EFH[b] per day (hours)**							
0-1	30.7 (6.8)	−1.6 (4.4)	−1.5 (5.8)	−1.7 (7.3)	−1.9 (6.6)	0	N/A
2-3	31 (7.4)	−3.3 (4.2)	−3.5 (5.8)	−2.3 (6.5)	−2.5 (7.7)	−2.301	.01
4-5	31.2 (6.8)	−5.3 (4.1)	−6.4 (6.4)	−5.7 (7.8)	−5.1 (8.5)	−4.325	<.001
6-7	31.8 (7.2)	−6.8 (4)	−8.9 (6.4)	−9.3 (7.4)	−8.1 (8.5)	−7.331	<.001
8-9	32.3 (7.5)	−8.4 (4.1)	−11.2 (6.7)	−12.5 (8.2)	−13.1 (11.1)	−9.251	<.001
≥10	33 (7.9)	−10.6 (5)	−14.8 (8)	−15.7 (12)	−15.7 (13.5)	−11.655	<.001

[a]N/A: not applicable.

[b]EFH: extended fasting hours.
